# Classification of regimes determining ultrasonic cavitation erosion in aqueous solutions containing dissolved air^☆^

**DOI:** 10.1016/j.ultsonch.2025.107324

**Published:** 2025-03-23

**Authors:** Dingkang Xia, Jianhua Wu, Kunpeng Su

**Affiliations:** a College of Water Conservancy and Hydropower Engineering, Hohai University, Nanjing 210098, China; b College of Hydraulic Science and Engineering, Yangzhou University, Yangzhou 225009, China

**Keywords:** Ultrasonic cavitation erosion, Dissolved air, Vaporous cavitation, Gaseous cavitation, Bubble formation

## Abstract

Ultrasonic cavitation is crucial for wastewater treatment, as it enhances the oxidation and degradation of contaminants. However, cavitation erosion of acoustic horn tips poses a significant challenge due to reduced treatment efficiency and increased operational costs. Since the air dissolved in liquids not only affects sonochemical yields but also complicates ultrasonic cavitation erosion, the quantitative analysis of dissolved air affecting cavitation erosion is required. This study experimentally investigated the effect of dissolved air on erosion by increasing the dissolved oxygen content from 1.18 to 18.30 mg·L^–1^ via pre-supersaturation and degasification methods. Employing ultrasonic vibrations, material removal results indicated that erosion was initially aggravated and then alleviated, with an increase in dissolved air shifting the state of the solution from an undersaturated to a supersaturated state. The most severe erosion was observed when the dissolved oxygen content reached 4 mg·L^–1^, which corresponded to a half-saturated state. Theoretical examinations of heterogeneous nucleation rates and energy dissipation following asymmetrical bubble collapse revealed four regimes: homogeneous nucleation, vaporous and gaseous cavitation bubble nucleation, and microbubble formation. With increasing dissolved air, accelerated vaporous cavitation aggravates erosion, while gaseous cavitation and microbubble formation alleviate erosion, which provides a classification of regimes determining cavitation erosion affected by dissolved air. These findings highlight the significant effect of dissolved air on ultrasonic cavitation erosion and, with a better understanding of these regimes, can aid in optimizing the design and operation of sonoreactors used for wastewater treatment.

## 1 Introduction

Acoustic cavitation refers to the formation, growth, and collapse of cavitation bubbles (CBs) in liquids irradiated with high-intensity ultrasound [1]. Although the severe damage caused to materials by cavitation is concerning [2], [3], [4], [5], acoustic cavitation plays an important role in various industries, including sonochemistry [6], [7], [8], [9]. Since the 1980 s, inexpensive and reliable ultrasonic generators such as piezoelectric horn-type reactors have made sonochemistry a promising and sustainable technology for wastewater treatment [10]. However, the cavitation erosion (CE) of ultrasonic horn tips can inhibit sonochemical reactions [11], and the frequent replacement of tips can increase operating costs [12]. Further, the presence of impurities, such as microparticles, microbubbles (MBs), and dissolved gases, significantly complicates the CE process [13], [14], [15], [16]. In wastewater treatment, it is necessary to pre-settle or filter particles from wastewater [17], while dissolved gases are generally left untreated. By controlling the pressure and temperature in sealed reactors or using gas sparging devices, the concentration of dissolved gases, particularly dissolved air (DA), can be precisely regulated. This is essential in practical applications, such as ultrasonication combined with biological treatment, and the content of DA (*φ*_DA_) merits attention [18]. Therefore, examining the influence of *φ*_DA_ on CE is imperative.

Prior research focused on the effect of total air content, including both air bubbles and DA, on the inception of cavitation. In the mid-1930s, a Venturi test revealed a relatively linear increase in the cavitation number with increasing total air content [19], indicating a lower probability of cavitation. However, contradictory findings subsequently emerged regarding whether increasing total air content would promote cavitation [20], [21], [22], [23]. Since DA could play a major role in promoting cavitation nucleation, there was an urgent need to present a detailed report on the role of entrained air bubbles and DA in inducing cavitation [24]. To this end, nonflowing test systems (i.e., vibratory apparatus) were utilized for distinguishing the cavitational effects of the two types of air [25], [26]. The results indicated that the cavitation threshold increased with a decrease in *φ*_DA_ [27].

With the advent of sonochemistry, research now focused on determining the effects of DA on sonoluminescence [28], sonochemiluminescence [29], and sonochemical yields [30]. Increasing *φ*_DA_ created more localized hotspots, which enhanced these sonochemical effects [28], [29], [30]. However, CE is induced by shockwaves or microjets generated during the asymmetrical collapse of CBs, and therefore, the effect of DA on CE differs from that on sonochemistry.

Experiments to determine the effect of air content on CE date back to 1937, with Mousson's Venturi tests indicating that an increase in the total air content reduced CE [31]. This trend was corroborated by numerous studies that used flowing tests [32], [33]. In 1969, Hobbs and Laird [34] pioneered experiments employing vibratory apparatus to demonstrate less severe CE at very low (∼10 % saturation) and near-saturated gas contents, with a CE peak under non-saturated conditions. A reduction in CE caused by degassing has also been reported [21]. The peak in CE and degassing-induced CE alleviation were verified in sonicated water, where the dissolved oxygen (DO) contents ranged from 4.06–6.34 mg·L^−1^ [35]. At extremely low air contents, it was hypothesized that further degassing could prevent cavitation and CE, whereas under supersaturation conditions, some studies suggested an alleviation of CE [36]. While the effect of DA on CE has garnered attention due to the advantages of DA in wastewater treatment, there has been limited quantitative research on the effect of varying DA saturation conditions. Thus, a comprehensive quantitative analysis of the relationship between CE and *φ*_DA_—specifically across a wider range of *φ*_DA_—is required to further investigate the underlying regimes.

Thus far, previous studies have attempted to explain the aggravating and alleviating effects of DA on CE. On the one hand, CE aggravation is linked to the intensified nucleation of vaporous cavitation bubbles (VBs), which is often mentioned in the discussion of cavitation inception and sonochemistry [27]. The nucleation rate increased with an increase in *φ*_DA_, because of the reduction in the surface tension of the liquid [37], [38], [39]. However, this reduction would also mitigate the asymmetrical collapse [40], and therefore, increasing the nucleation rate could have less effect on CE compared to that on sonochemistry. On the other hand, CE alleviation suggests another dominant mechanism. One study reported that increasing the amount of non-condensable gas in CBs reduced the rate of CB collapse [41]. Gaseous cavitation, which is less erosive than vaporous cavitation, might become more significant with a higher dissolved gas content [42]. In addition, because of the variation in pressure, the dissolved gases could form MBs [43], which could relieve local negative pressures, damp shockwaves, and deflect microjets [44], [45], [46]. This mechanism underlies the installation of aeration devices in hydraulic engineering [47]. Although several possible mechanisms were proposed in various studies, the predominant regimes at various *φ*_DA_ levels remain to be fully determined to clarify the effects of DA on CE.

This paper aims to investigate the effect of *φ*_DA_ on CE and elucidate the relevant mechanisms. To this end, aqueous solutions with different saturation conditions were prepared through pre-supersaturation and degasification to obtain a wider range of stable *φ*_DA_ values. Vibratory CE tests were conducted in these solutions. Based on the results of specimen surface deterioration and the theoretical analysis, the underlying regimes were discussed and classified.

## 2 Materials and methods

### 2.1 Variation of *φ*_DA_

In this study, the concentration of DO (*φ*_DO_) was used as an indicator of *φ*_DA_ because of the stable partial pressure of oxygen in the atmosphere. *φ*_DO_ was measured using a DO meter (YSK-607A-3 M, Shanghai Yuwo Equipment Co., Ltd.), whose electrode has been polarized and calibrated for zero and full oxygen levels before tests. During the measurement, the electrode was immersed in the solution and gently swayed to obtain a stable reading. At 25 °C and 1 atm, the theoretical solubility of oxygen in water is 8.26 mg·L^–1^, whereas under the same conditions in the lab, it was measured as 8.17 ± 0.50 mg·L^–1^ in double-distilled water aerated with fine bubbles for 1 h.

**Fig. 1:**
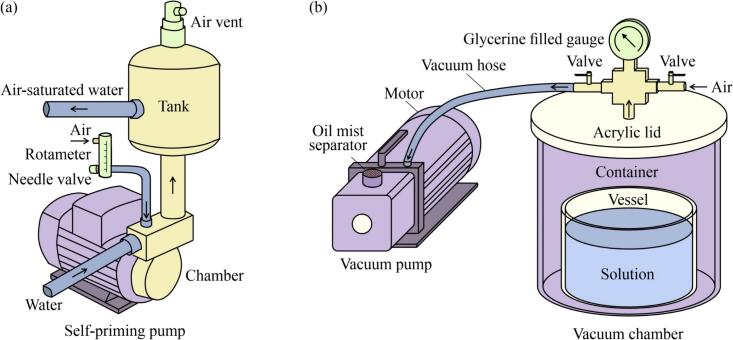
Schematic of (a) a pre-supersaturation system for forming a supersaturated mixture of air and water, and (b) a degasification system for decreasing the dissolved air content in the aqueous solution without heating.

Two systems were employed to achieve various *φ*_DA_ values in aqueous solutions without altering the temperature: one for pre-supersaturation and the other for degasification (Fig. 1). The pre-supersaturation system (Fig. 1a) was designed to form a supersaturated mixture of air and water, which was previously employed by the authors to generate micron-sized air bubbles with additional depressurization [48]. In this study, double-distilled water with a conductivity of 0.036 mS·m^−1^ was used, which was supplied by Yangzhou Zhongken Pure Water Equipment Co., Ltd. The core component of this system was a self-priming centrifugal pump (550 W; Dongguan Yaoshun Spray Technology Co., Ltd.). A rotating impeller in the pump chamber could dissolve air due to the reduced pressure created by fluid recirculation. The air flow rate was controlled using a needle valve and measured using a rotameter (LZB-3WB, Darhor Technology Co., Ltd.). Then, the mixture was pumped into a tank. After the excess undissolved air in the tank was relieved through an automatic air vent, the internal pressure was stabilized, and the supersaturated solutions were prepared.

After the pre-supersaturation stage, the vacuum degasification system (Fig. 1b) was employed to reduce *φ*_DA_ without heating because the dissolved gas is less soluble under reduced pressure. This system included a vacuum pump (RS-1, Wenling Hongbaoshi Vacuum Equipment Co., Ltd.) and a stainless-steel chamber (diameter = 25 cm and height = 20 cm). The pump evacuated air from the chamber through a vacuum hose connected to an outlet valve. An intake valve balanced the internal and external pressures to open the chamber. A transparent acrylic lid was placed for sealing and visibility into the chamber. A glycerin-filled vacuum gauge (−0.1–0 MPa) installed on the lid enabled monitoring the internal pressure during degasification. The pressure inside the chamber decreased when the intake valve was closed and the outlet valve was opened, thereby allowing DA in the solution to escape in the form of bubbles.

**Fig. 2:**
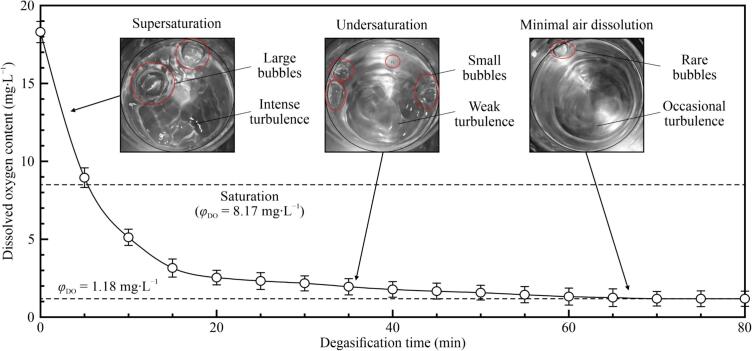
Dissolved oxygen content (*φ*_DO_) as a function of degasification time for vacuum degasification of pre-supersaturated aqueous solutions, with the temperature maintained at 25 ± 1 °C. Error bars indicate the standard deviation. The dashed lines represent the saturated and minimum *φ*_DO_ values obtained in this study. The three images show the solution surfaces under conditions of supersaturation, undersaturation and minimal-air-dissolution.

In the gas supersaturation system, the flow rates of water and air were maintained at 10 ± 1 L·min^−1^ and 100 ± 10 mL·min^−1^, respectively. Under these conditions, *φ*_DO_ of the supersaturated solutions was 18.30 ± 0.50 mg·L^–1^. Immediately after pre-supersaturation, the solution was transferred to the vessel in the vacuum degasification system. The degasification was performed for 90 min, with the solution temperature maintained at 25 ± 1 °C using an immersion heater rod. The effect of degasification time on *φ*_DO_ was measured at 5-min intervals (Fig. 2).

Before degasification, DA in solutions was supersaturated. It was released in the form of large bubbles (1–3 cm in diameter) under the reduced chamber pressure, causing intense turbulence. After 10-min degasification, *φ*_DO_ dropped to 5.11 ± 0.50 mg·L^–1^, indicating undersaturation. Thereafter, the rate of degassing decreased gradually, accompanied by smaller bubbles and weaker turbulence. When the total degassing time reached 20 min, *φ*_DO_ was halved compared to the value at 10 min, with a degassing rate of approximately 0.08 mg·L^–1^·min^−1^. The degassing was considered complete before 70 min, as rare bubbles and occasional surface turbulence were observed.

However, after the lid was removed, the rebalance of the internal and external pressures caused some atmospheric air to dissolve back into the solution. *φ*_DO_ was measured to be 1.18 ± 0.50 mg·L^–1^ immediately following pressure rebalance, and it increased by 0.46 mg·L^–1^ after the solution was left undisturbed for 5 min. Therefore, the degasification time was used to adjust *φ*_DO_ within the range of 1.18–18.30 mg·L^–1^. To investigate the effects of *φ*_DA_ on CE, the degasification time was obtained from Fig. 2 based on the interpolation approach. All the experimental cases are listed in Table 1.

**Table 1:** Experimental cases and required degasification times.

Case No.	Dissolved oxygen content (mg·L^–1^) *	Degasification time (min) *	Case No.	Dissolved oxygen content (mg·L^–1^) *	Degasification time (min) *
1	1.18	70.0	6	10.00	4.2
2	2.00	33.6	7	12.00	3.1
3	4.00	12.4	8	14.00	2.0
4	6.00	8.2	9	16.00	1.1
5	8.00	5.7	10	18.30	0

* The allowable deviations of dissolved oxygen content and degasification time were 0.50 mg·L^–1^ and 5 s, respectively.

### 2.2 Cavitation erosion experiment

**Fig. 3:**
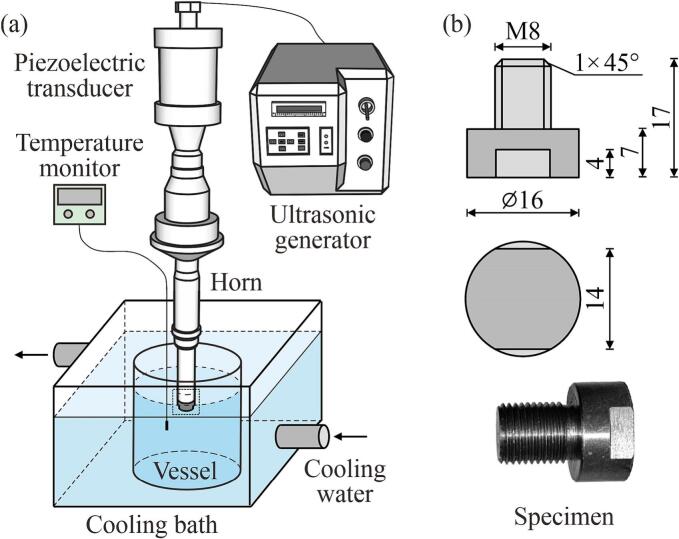
(a) Schematic of the experimental set-up, and (b) dimensions and photograph of a test specimen (mm).

Fig. 3 shows the vibratory facility and specimens used in the experiments. Accelerated CE tests were performed using a piezoelectric device (VCY–1500, Shanghai Permanent Ultrasonic Equipment Co. Ltd.) that included an ultrasonic generator (600 W), a piezoelectric transducer, and an amplifying horn (Fig. 3a). The transducer generated axial oscillations at a frequency of 20 kHz, and the peak-to-peak amplitude of the horn tip was 50 μm. Each specimen was tightly threaded onto the horn tip, and a light coating of silicone grease was applied to enhance the coupling. Further, specimens made from AISI 1045 carbon steel were machined from the same bar stock to ensure consistent mechanical properties and chemical compositions (Fig. 3b). Before the test, the test surfaces were ground and polished to an arithmetic average surface roughness (*R*_a_) of 20 nm.

All tests were conducted centrally with the specimen submerged at a depth of 10 mm in a cylindrical vessel. The vessel was positioned within a rectangular outer glass tank filled with a cooling bath to maintain a stable solution temperature of 25 °C near the surface of the specimen. The temperature monitoring probe was located 3 mm in the radial direction from the specimen periphery and 3 mm below the specimen surface, to avoid interference with the cavitation process. To obtain a history of mass loss versus time, each test lasted 300 min, with the specimen removed at 30-min intervals. After each removal, the specimen was ultrasonically rinsed, dried, cooled, and weighed using an analytical balance with an accuracy of ±0.1 mg (FA1004, Shanghai Hengji Scientific Instrument Co., Ltd). Each test was repeated three times, and the average values were used for statistical reliability.

The test liquid (double-distilled water dissolved with air) was replaced each time before reinstalling the specimen considering the potential variation in *φ*_DA_ caused by ultrasonic degassing or air dissolving. The effect of 30-min ultrasonic irradiation on *φ*_DO_ was carefully examined. For the lowest initial *φ*_DO_ (Case 1), *φ*_DO_ increased by ∼0.74 mg·L^–1^ after 30-min irradiation. A plausible explanation could be that the air, which was dissolved from the atmosphere due to the concentration gradient, was degassed, and the degassing effect was enhanced at our ultrasonic power and frequency [49]. For Case 10 with supersaturated air, the initial *φ*_DO_ was decreased by only 9.6 % after 30-min irradiation, which is consistent with the decreasing rate of *φ*_DO_ attributed to sonication reported in a recent study [50]. The observed reduction in *φ*_DO_ was acceptable in this study.

## 3 Results and discussion

### 3.1 Effect of DA on CE

**Fig. 4:**
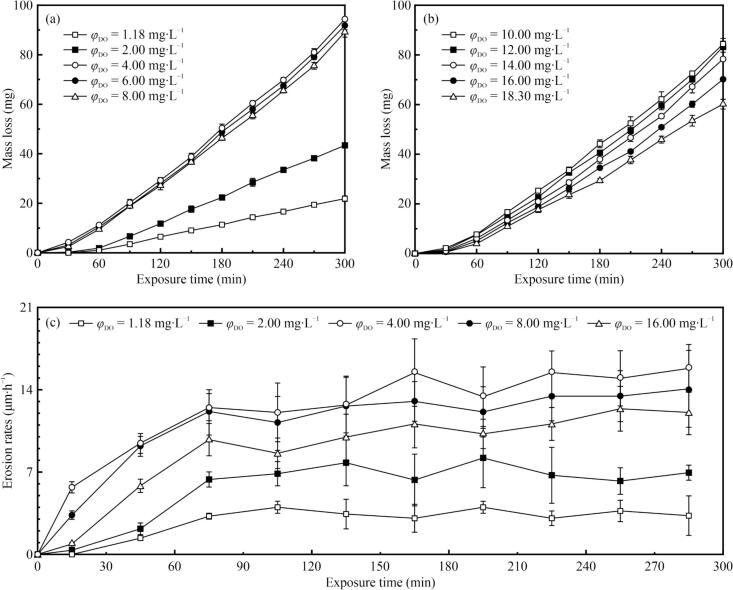
Measured mass loss of specimens as a function of exposure time in (a) undersaturation cases (dissolved oxygen content *φ*_DO_ = 1.18–8.00 mg·L^–1^), and (b) supersaturation cases (*φ*_DO_ = 10.00–18.30 mg·L^–1^). (c) Erosion rates as a function of exposure time in representative cases. Error bars indicate one standard deviation and are displayed when they are larger than the data point markers.

Fig. 4 shows the experimental data for the undersaturation and supersaturation cases listed in Table 1. The erosion rates were determined from the change between two consecutive mean depths of erosion (*MDE*s, calculated by dividing the measured mass loss by the material density and by the specimen surface area). As shown in Fig. 4(a) and (b), the measured mass losses of the test specimens increased with exposure time in all cases. The erosion rates (Fig. 4c) revealed that some samples exhibited incubation periods within the initial 30 min of exposure. Following the incubation period, the mass loss accelerated and then increased by a similar amount every 30 min, suggesting a nearly constant rate after the acceleration period. It is also indicated that CE achieved its maximum rate after 60 min of exposure, without distinct deceleration stages observed. These results paralleled our previous findings obtained with distilled water containing glass beads or sediments with particle diameters ranging from 10 to 100 μm [13], [14], [15].

Under undersaturation conditions (Fig. 4a), the case with *φ*_DO_ = 1.18 mg·L^–1^ was considered as a minimal-air case, while *φ*_DO_ = 8.00 mg·L^–1^ represented near-saturation. The mass loss at 300 min in the minimal-air case was less than those in other cases. Specifically, the mass losses after 120 min in all cases of undersaturation, except in the case with *φ*_DO_ = 2.00 mg·L^–1^, surpassed the loss at 300 min in the minimal-air case. When *φ*_DO_ = 2.00 mg·L^–1^, the mass loss was nearly double that in the minimal-air case; however, it was still less than half of the losses observed at *φ*_DO_ = 4.00–8.00 mg·L^–1^ for the same exposure duration. When *φ*_DO_ was in the range of 4.00–8.00 mg·L^–1^, the mass loss at *φ*_DO_ = 4.00 mg·L^–1^ (supposed to be half saturation) was the most (94.4 mg) and it decreased slightly with increasing *φ*_DO_, with a small difference of less than 6 mg at 300 min within this range.

Under supersaturation conditions (Fig. 4b), significant differences were observed in the way the mass loss varied with *φ*_DO_. The mass loss at 300 min decreased with increasing *φ*_DO_ in the range of 10.00–18.30 mg·L^–1^; however, it was less than that at *φ*_DO_ = 8.00 mg·L^–1^ (89.2 mg). At *φ*_DO_ = 10.00 mg·L^–1^, the mass loss at 300 min was 95 % of that in the near-saturation case, and 1.4 times that at *φ*_DO_ = 18.30 mg·L^–1^. Further, the decrease in mass loss at 300 min with increasing *φ*_DO_ in the supersaturated solutions was more pronounced than those in the cases of lower *φ*_DO_. At *φ*_DO_ = 14.00 mg·L^–1^, the mass loss at 300 min was 7 % less than that at *φ*_DO_ = 10.00 mg·L^–1^, yet 30 % more than that at *φ*_DO_ = 18.30 mg·L^–1^. Although the least mass loss under supersaturation conditions occurred at *φ*_DO_ = 18.30 mg·L^–1^, it was still nearly two times higher than that in the minimal-air case.

We defined the percentage change in the mass loss as Δ*m*/*m*_s_ to better characterize the effects of DA on CE; here, Δ*m* and *m*_s_ represent the variation in the cumulative mass loss from the near-saturation case to an undersaturation or supersaturation case, and the cumulative mass loss under the near-saturation condition, respectively. This parameter can be either positive (aggravating CE) or negative (alleviating CE), with Δ*m*/*m*_s_ = 0 indicating no difference in the mass loss between the near-saturation case and another case. Here, *α* represents the relative dissolved air content (ratio of actual gas content to saturated gas content).

**Fig. 5:**
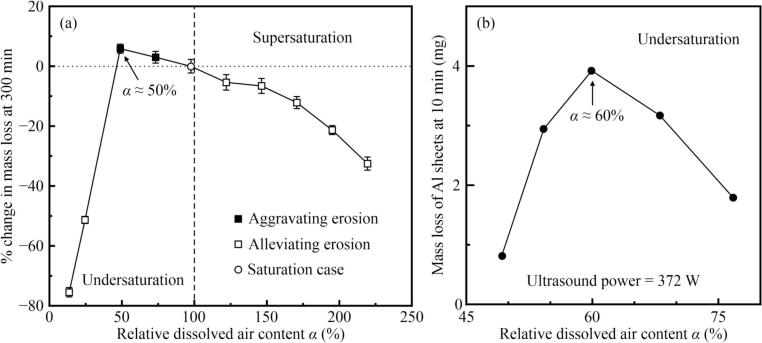
(a) Percentage change in mass loss at 300 min, and (b) mass loss of aluminum (Al) sheets sonicated for 10 min at 372 W [35], plotted against the relative dissolved air content α. The dotted line represents no change in mass loss while the dashed line represents saturated gas content.

Fig. 5(a) depicts the relationship between Δ*m*/*m*_s_ and *α*, which indicates a clear distinction between the undersaturation and supersaturation cases. Compared to the saturation case, degassing initially aggravated the CE, with the most severe CE occurring under half-saturation conditions; however, further degassing significantly alleviated the CE. Conversely, when the gas was supersaturated, CE was alleviated with increasing *α*, at a higher rate. These results indicated that CE was aggravated only when *α* was approximately in the range of 50 %–100 %. Although CE was alleviated under both supersaturated and undersaturated conditions, the alleviation effect at *α* ≈ 200 % was weaker than that at *α* ≈ 25 %.

Fig. 5(b) presents the experimental results for the aluminum sheets tested for 10 min at 372 W [35]. The experiments were conducted in undersaturated solutions, and therefore, no Δ*m*/*m*_s_ values were obtained. Although direct comparisons are challenging because of differences in the specimen materials, some similarities are evident. Notably, both studies showed an initial increase in mass loss, followed by a decrease in undersaturated solutions, with a peak CE occurring when *α* ranged from 50 %–60 %. However, when *α* exceeded 60 %, their findings indicated a more pronounced reduction in CE compared to that in this study. This discrepancy may be attributed to several factors, including a shorter exposure time, a more pliable nature of the aluminum sheets, and a lower ultrasound power used in their experiments, which could lead to a prolonged incubation period and reduced CE. Despite these differences, their results were consistent with our findings.

**Fig. 6:**
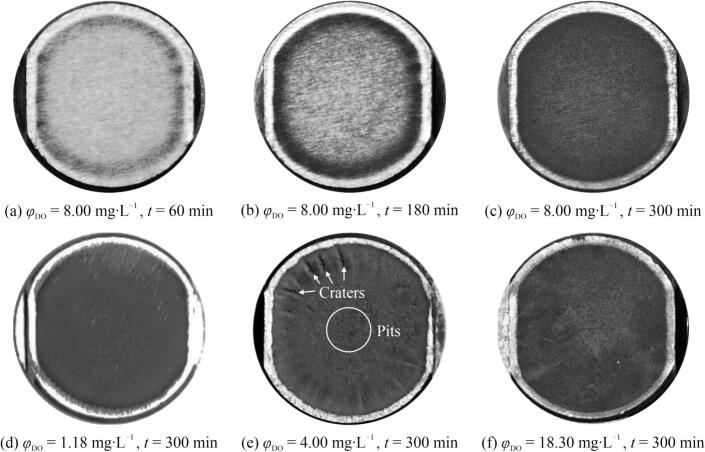
Photographs of the eroded surfaces of test specimens after various exposure time (*t*) in aqueous solutions containing different dissolved air content (*φ*_DO_).

In our study, the mass loss at 300 min initially increased (*α* < 50 %) and then decreased (*α* > 50 %) with increasing *α*, which implied that minimal DA resulted in minimal mass loss, whereas increasing the DA initially increased the mass loss. However, beyond a certain *α*, further increases reduced the mass loss. Fig. 6 provides the macromorphologies of the representative specimen surfaces under different exposure times and *φ*_DO_ conditions to further differentiate the effects of varying *φ*_DO_ on CE.

In the near-saturation case, the eroded region was predominantly centered at the middle of the specimen surfaces, whereas the outermost edge retained a metallic luster, indicating minimal CE and suggesting an almost uneroded region (Fig. 6a–c). Initially, the CE appeared on the periphery of the eroded region (Fig. 6a) and progressed towards the center (Fig. 6b). After completion of the exposure, the eroded region lost nearly all metallic luster and was dotted with small pits (Fig. 6c). This surface pattern was generally consistent across all cases; however, there were marked differences in the surface morphology with different *φ*_DO_.

In the minimal-air case, few pits were observed in the surface center, and the eroded region did not entirely lose its luster (Fig. 6d). At half-saturation, the CE was the most pronounced (Fig. 6e), featuring deep radial craters encroaching on and narrowing the uneroded region. The small pits were more concentrated in the center and deeper than those in the near-saturation case (Fig. 6c) despite only a slight difference in mass loss between these two conditions (Fig. 4a). Distinct craters were absent in the supersaturation case (Fig. 6f). The pits were more uniformly distributed but smaller and shallower than those in the near-saturation case.

The photographic results of the eroded surfaces closely aligned with the mass loss results; however, the differences in the eroded surfaces were significant even when the mass losses exhibited minor variations. This implies that under different saturation conditions, the regimes influencing the CE may vary although they have not been well elucidated in the existing literature. Given the complexity of the underlying regimes, a more comprehensive classification model would be proposed.

### 3.2 Effect of VB nucleation on CE

The initial stage of CB formation, known as nucleation, plays a critical role in determining both cavitation intensity and CE. Nucleation processes are typically categorized as either homogeneous or heterogeneous. Theoretically, cavitation should not occur in ultrapure liquids with high cavitation thresholds because of the lack of surfaces for heterogeneous nucleation. However, container walls and impurities such as DA can provide such surfaces. Early studies demonstrated that the introduction of DA significantly facilitated nucleation compared to homogeneous nucleation [51]. Therefore, heterogeneous nucleation is a key factor influencing CE in undersaturated solutions containing DA.

Heterogeneous nucleation in sonicated liquids containing DA commonly involves VB and gaseous cavitation bubble (GB) nucleation processes. When the local static pressure of the liquid decreases below the saturated vapor pressure, VBs form and subsequently collapse rapidly if the pressure exceeds the saturated vapor pressure. This process, known as vaporous cavitation, generates shockwaves and microjets during collapse, thereby contributing to CE. Therefore, it is important to examine the effects of DA on VB nucleation.

According to the Classical Nucleation Theory [52], the nucleation rate of VBs per unit volume is given by

(1) $$J=ZDN\text{exp}\left(-\frac{\Delta G^{*}}{k_{\text{B}}T}\right),$$

where *Z* represents the Zeldovich factor, which defines the probability of nucleation versus dissolution. Further, *D*, *N*, ΔG*, *k*_B_, and *T* represent the molecular attachment rate to the nucleus, number of nucleation sites, free energy barrier governing nucleation, Boltzmann constant, and absolute temperature, respectively. The probability of a nucleus forming at a given site increases exponentially with (−ΔG*/*k*_B_*T*), thereby highlighting the critical role of ΔG* in nucleation at a constant temperature.

The free energy of homogeneous nucleation Δ*G*_homo_ can be expressed as the sum of bulk and surface terms.

(2) $$\Delta G_{\text{homo}}=-\frac{4}{3}\pi r^{3}\Delta g_{\text{v}}+4\pi r^{2}\sigma ,$$

where *r*, Δ*g*_v_, and *σ* represent the radius of a nucleus assumed to be spherical, difference in free energy per unit volume between the nucleating phase and the surrounding thermodynamic phase, and surface tension coefficient of the surrounding phase, respectively.

For heterogeneous nucleation, the effective surface area is smaller than that for homogeneous nucleation because part of the nucleus boundary is affected by impurities such as DA. The free energy of the heterogeneous nucleation can be expressed as

(3) $$\Delta G_{\text{hetero}}=f\left(\theta \right)\Delta G_{\text{homo}}=\frac{2-3\cos\theta +\cos^{3}\theta }{4}\Delta G_{\text{homo}},$$

where *f*(*θ*) represents a geometric factor introduced to account for the interface characteristics and is determined by the contact angle *θ* [52].

For small nuclei, the surface term dominates, which makes Δ*G*_homo_ positive, while for sufficiently large nuclei, the bulk term prevails. Therefore, a critical radius r* exists where the free energy reaches its maximum. The mathematical expressions for r* and the corresponding energy barrier ΔG*_homo_ are given by

(4) $$r^{*}=\frac{2\sigma }{\left|\Delta g_{\text{v}}\right|},$$

and

(5) $$\Delta G_{\text{homo}}^{*}=\frac{4\pi \sigma {\left(r^{*}\right)}^{2}}{3}=\frac{16\pi \sigma^{3}}{3{\left(\Delta g_{\text{v}}\right)}^{2}}.$$

These equations indicate that ΔG*_homo_ depends strongly on *σ*. Although the precise effect of DA on cavitation intensity remains challenging to predict, studies have shown that nucleation could accelerate with increased *φ*_DA_ due to the reduction in surface tension [38]. Thus, *σ* can serve as a key indicator for evaluating the effect of DA on the nucleation rate. Assuming that variations in Δ*g*_v_ and *ZDN* are negligible, the energy barrier for heterogeneous nucleation ΔG*_hetero_ and the ratio of the heterogeneous to homogeneous nucleation rates *R*_h_ can be derived as

(6) $$\Delta G_{\text{hetero}}^{*}=\frac{{16\pi \text{(}\sigma \prime )}^{3}}{3{\left(\Delta g_{\text{v}}\right)}^{2}}f\left(\theta \right),$$

and

(7) $$R_{\text{h}}=\exp\left(\frac{\Delta G_{\text{homo}}^{*}-\Delta G_{\text{hetero}}^{*}}{k_{\text{B}}T}\right)=\exp\left\{\frac{16\pi }{3{\left(\Delta g_{\text{v}}\right)}^{2}k_{\text{B}}T}\left[\sigma^{3}{-\text{(}\sigma \prime )}^{3}f\left(\theta \right)\right]\right\},$$

where *σ*′ represents the surface tension coefficient of the liquid containing DA. Based on the findings of r* (≈1 nm) [53], Δ*g*_v_ was estimated to be ∼144 MPa, calculated using Eq. (4).

**Fig. 7:**
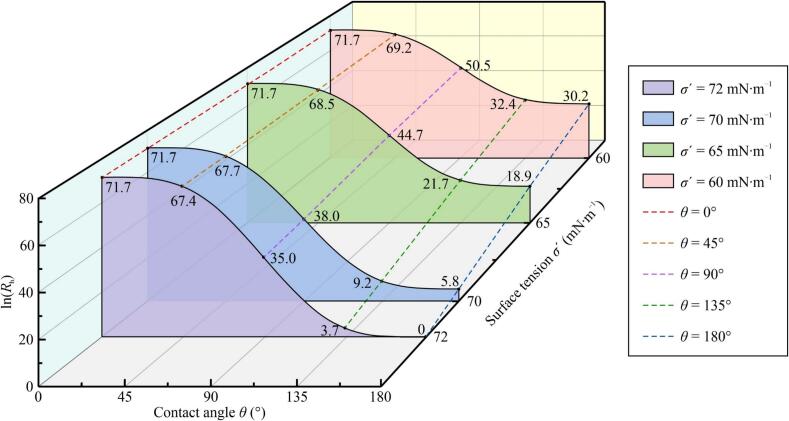
Ratio of heterogeneous nucleation rate to homogeneous nucleation rate (*R*_h_) as a function of contact angle (*θ*) and surface tension in the liquid containing dissolved air (*σ*′). The filled zones represent the effect of *θ* at a fixed *σ*′, whereas the colored dashed lines illustrate the effect of *σ*′ at a fixed *θ*. The labeled numbers represent the calculated values of ln(*R*_h_). Calculations were performed with Δ*g*_v_ = 144 MPa, *k*_B_ = 1.38 × 10^−23^ J·K^−1^, *T* = 298.15 K and *σ* = 72 mN·m^−1^.

According to Eq. (7), *R*_h_ primarily depends on *θ* and *σ*′, assuming that Δ*g*_v_ and *T* remain constant. Fig. 7 presents a graph of ln(*R*_h_) as a function of *θ* and *σ*′, indicating that ln(*R*_h_) is significantly increased as both *θ* and *σ*′ decrease. When *θ* = 180° and *σ*′ = *σ*, *R*_h_ = 1, which implies that the nucleation resembles the homogeneous behavior. A sharp increase in *R*_h_ occurs as *θ* decreases; for example, when *θ* decreases to 90°, *f*(*θ*) becomes 1/2 and *R*_h_ reaches 10^15^ while the surface tension remains unchanged. A further reduction in *θ* from 90° to 0° slows the increase in *R*_h_, resulting in smaller variations in *R*_h_ as *σ*′ changes. At *θ* = 45°, ln(*R*_h_) at *σ*′ = *σ* exceeds 97 % of that at *σ*′ = 60 mN·m^−1^, while ln(*R*_h_) at *σ*′ = 70 mN·m^−1^ is nearly 2.5 times that at *σ*′ = *σ* when *θ* = 135°. Even in cases approaching homogeneous nucleation, a slight reduction in *σ*′ can greatly accelerate nucleation: a mere 3 % decrease in *σ*′ can increase *R*_h_ to ∼330. Moreover, it appears that ln(*R*_h_) is linearly proportional to *σ′* at a fixed *θ*. For example, when *θ* = 90°, ln(*R*_h_) ≈ −1.29*σ′* + 128.1, and the linear correlation coefficients for all five colored dashed lines exceed 0.997. Although this linear relationship might not be physically meaningful, it might offer a convenient method for estimating *R*_h_ and the nucleation rate.

Previous research revealed that increasing *φ*_DA_ could decrease both *σ*′ (up to 20 %) and *θ* (to 44°) [54]. According to our analysis, these changes can lead to a substantial increase in *R*_h_ and the acceleration of heterogeneous nucleation, which can explain the observed increase in Δ*m*/*m*_s_ with the initial introduction of DA and increasing *φ*_DA_ (Fig. 5a). The appearance of deep radial craters (Fig. 6e) can also be attributed to the intensified VB nucleation, which induces a stronger impact of shockwaves or microjets at the specimen surfaces. However, a further increase in *φ*_DA_ resulted in a reduction in Δ*m*/*m*_s_ (Fig. 5a) and the disappearance of craters (Fig. 6c), suggesting that another regime could alleviate CE.

### 3.3 Effect of GB nucleation on CE

Similar to VB nucleation, GB nucleation is also a heterogeneous process. This involves the diffusion of non-condensable gases dissolved in a liquid to form GBs with a decrease in pressure as per Henry’s Law. The GBs will redissolve with an increase in pressure. This process is known as gaseous cavitation. Both vaporous and gaseous cavitation can produce noise, high energy with elevated temperatures, and oxidation; however, gaseous cavitation generally causes less severe or even no CE [55]. It is hypothesized that the dominance of GB nucleation contributes to the alleviation of CE. Thus, the distinction between VB and GB nucleation influenced by *φ*_DA_ warrants further investigation.

Two modified models based on Classical Nucleation Theory described both VB [56] and GB nucleation [57] in solutions containing DA. In these models, the surface energy was recognized as a crucial factor. During VB nucleation, the surface energy involves the disruption of molecular clusters from the surrounding liquid, which is closely linked to the intermolecular potential energy [58]. Conversely, GB nucleation is primarily governed by the translational motion energy of the gas molecules, which is constrained during dissolution by the solvent–solute interactions.

According to these models, the GB and VB nucleation rates per unit volume can be expressed as [56], [57]

(8) $$J_{\text{GB}}=Z_{\text{GB}}D_{\text{GB}}N_{\text{GB}}\text{exp}\left(-\frac{1}{2}n_{\text{GB}}^{\frac{2}{3}}\right),$$

and

(9) $$J_{\text{VB}}=Z_{\text{VB}}D_{\text{VB}}N_{\text{VB}}\text{exp}\left(-\frac{1}{2}n_{\text{VB}}^{\frac{2}{3}}\right),$$

where *n* denotes the number of molecules in a critical cluster that is the smallest molecular cluster at an unstable equilibrium during nucleation. The subscripts VB and GB denote vaporous and gaseous cavitation bubbles, respectively.

The ratio of GB to VB nucleation rate is given by

(10) $$R_{\text{GV}}=\frac{J_{\text{GB}}}{J_{\text{VB}}}=\frac{Z_{\text{GB}}D_{\text{GB}}}{Z_{\text{VB}}D_{\text{VB}}}\frac{N_{\text{GB}}}{N_{\text{VB}}}\text{exp}\left(\frac{1}{2}n_{\text{VB}}^{\frac{2}{3}}-\frac{1}{2}n_{\text{GB}}^{\frac{2}{3}}\right).$$

According to Refs. [56], [57], *Z*_GB_*D*_GB_ and *Z*_VB_*D*_VB_ are proportional to *N*_GB_ and *N*_VB_, respectively, and both are affected by solution properties. Assuming that DA acts as a molecular impurity and that the solution properties remain unchanged, *Z*_GB_*D*_GB_/*Z*_VB_*D*_VB_ can be expressed as

(11) $$\frac{Z_{\text{GB}}D_{\text{GB}}}{Z_{\text{VB}}D_{\text{VB}}}=\frac{N_{\text{GB}}}{N_{\text{VB}}}\frac{\beta_{\text{GB}}}{\beta_{\text{VB}}}{\left(\frac{m_{\text{GB}}}{m_{\text{VB}}}\right)}^{-\frac{1}{2}}{\left(\frac{v_{\text{GB}}}{v_{\text{VB}}}\right)}^{\frac{2}{3}},$$

where *β*, *m*, and *v* represent the accommodation coefficient of molecules, molecule mass, and molecule volume, respectively. *R*_GV_ simplifies to

(12) $$R_{\text{GV}}=\frac{\beta_{\text{GB}}}{\beta_{\text{VB}}}{\left(\frac{m_{\text{GB}}}{m_{\text{VB}}}\right)}^{-\frac{1}{2}}{\left(\frac{v_{\text{GB}}}{v_{\text{VB}}}\right)}^{-\frac{4}{3}}\varphi_{\text{DA}}^{\text{2}}\text{exp}\left(\frac{1}{2}n_{\text{VB}}^{\frac{2}{3}}-\frac{1}{2}n_{\text{GB}}^{\frac{2}{3}}\right)=k\varphi_{\text{DA}}^{\text{2}},$$

where *k* represents a factor corresponding to the properties of gas and vapor molecules, independent of *φ*_DA_.

For subsequent calculations, *β*_GB_ and *β*_VB_ were both assumed to be unity. For air and water, *m*_GB_/*m*_VB_ was 29/18. Further, *v*_GB_ and *v*_VB_ were estimated as 3.72 × 10^−2^ nm^3^ and 2.99 × 10^−2^ nm^3^, respectively, at standard temperature and pressure according to the ideal gas law. Using critical clusters with r* (≈1 nm), *n*_GB_ and *n*_VB_ were ∼140 and 112, respectively. Thus, *k* was calculated to be ∼11, which indicated that the variation in *φ*_DA_ would significantly influence *R*_GV_. Although previously treated as impurities, DA molecules participated in both nucleation processes. For example, when *φ*_DA_ is sufficiently small, the introduced DA can lower the energy barrier and act as nuclei for VB formation while GB nucleation may be negligible. However, GB nucleation accelerates more rapidly than VB nucleation when *φ*_DA_ increases beyond a certain threshold. Further, this indicates an applicable range of *φ*_DA_ in Eq. (12).

**Fig. 8:**
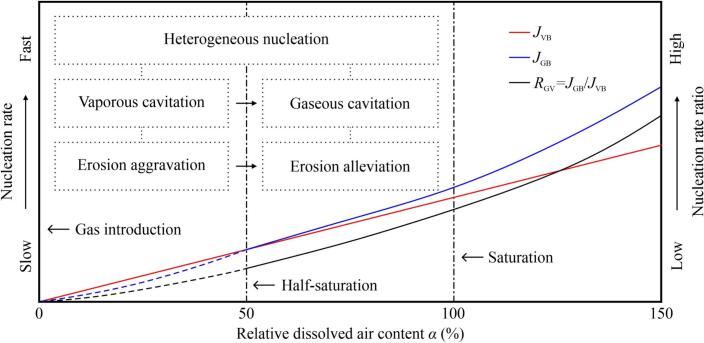
Nucleation rates of vaporous (*J*_VB_) and gaseous (*J*_GB_) cavitation bubbles against relative dissolved air content *α*. The dot-dashed lines represent various saturation conditions. The solid curves depict two nucleation rates and their ratio (*R*_GV_), whereas the dashed curves indicate that they are not applicable within this range of saturation. All intersections among these curves and lines are for illustrative purposes only.

Theoretical relationships between *J*_GB_, *J*_VB_, *R*_GV_, and *α* are illustrated in Fig. 8. Without dissolved gas, homogeneous nucleation faces a high free-energy barrier, preventing CE. Once gas is introduced into the liquid, the barrier is reduced because of molecular attraction, facilitating the liquid–gas transition and heterogeneous nucleation. *J*_VB_ increases rapidly because of the reduced barrier, which leads to an aggravated CE. However, more DA molecules act as GB nuclei as *α* increases, thereby increasing *J*_GB_. Although both *J*_GB_ and *J*_VB_ increase with increasing *α*, gaseous cavitation accounts for a larger proportion. Furthermore, the presence of DA in clusters and VBs can slow the growth and collapse, reducing the energy of shockwaves or microjets [59]. Consequently, the CE was aggravated and then alleviated with an increase in *α*, even though both rates increased.

The above analysis suggests a correlation between the two types of nucleation and CE in the undersaturated solutions. As shown in Fig. 4b, the predominant gaseous cavitation can still explain the alleviation trend even under supersaturation. However, a noticeable difference was observed in the eroded surfaces between the supersaturated (Fig. 6f) and saturated (Fig. 6c) conditions. Under supersaturation conditions, the specimens exhibited more uniformly distributed pits and fewer pronounced craters, suggesting that DA may indirectly affect CE. This effect appears to stem from modifications in the impact dynamics of shockwaves or microjets on surfaces, rather than changes in the intensity of vaporous or gaseous cavitation.

### 3.4 Effect of MB formation on CE

**Fig. 9:**
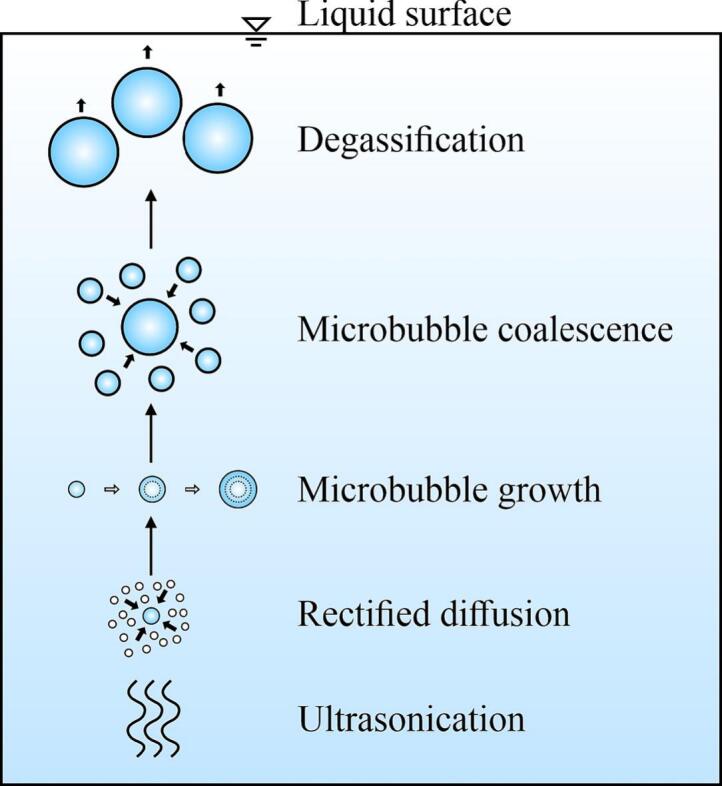
Schematic of microbubble formation during ultrasonication. White solid circles represent dissolved gas molecules and blue-white gradient circles represent microbubbles. (For interpretation of the references to colour in this figure legend, the reader is referred to the web version of this article.)

Under supersaturation conditions, large amounts of visible and stable MBs (different from CBs) can be formed during ultrasonication. As depicted in Fig. 9, the origin of these MBs was DA caused by ultrasonic degassing [49]. Ultrasonic waves create alternating positive and negative pressure phases: during the negative phase, near-vacuum MBs form because of intermolecular adhesion forces overcome by ultrasonic waves. During the positive phase, the gas inside the MBs will dissolve in the liquid or the MBs will collapse. However, in the presence of ultrasound, DA is forced to diffuse into the MBs, which is termed rectified diffusion [60]. This diffusion can expand MBs instead of collapsing them. The larger surface area of the expanded MBs facilitate gas absorption. In an ultrasonic field, these MBs are well distributed and may coalesce because of the secondary Bjerknes force [61], further increasing their size. These MBs will rise and gather around the specimen surfaces or escape the liquid.

The efficiency of MB formation depends not only on the frequency, amplitude, and power of the ultrasound, but also on *φ*_DA_. Under undersaturation conditions, MBs will dissolve in the liquid due to concentration gradients, and ultrasonication can even cause gas absorption into the liquid [62]. Conversely, higher *φ*_DA_ accelerates ultrasonic degassing and MB formation, which was observed during the experiments as more MBs forming at higher *φ*_DA_. This was also why the test solutions were renewed every 30 min. Consequently, the formed MBs should have a greater effect on alleviating CE in supersaturated solutions than DA.

**Fig. 10:**
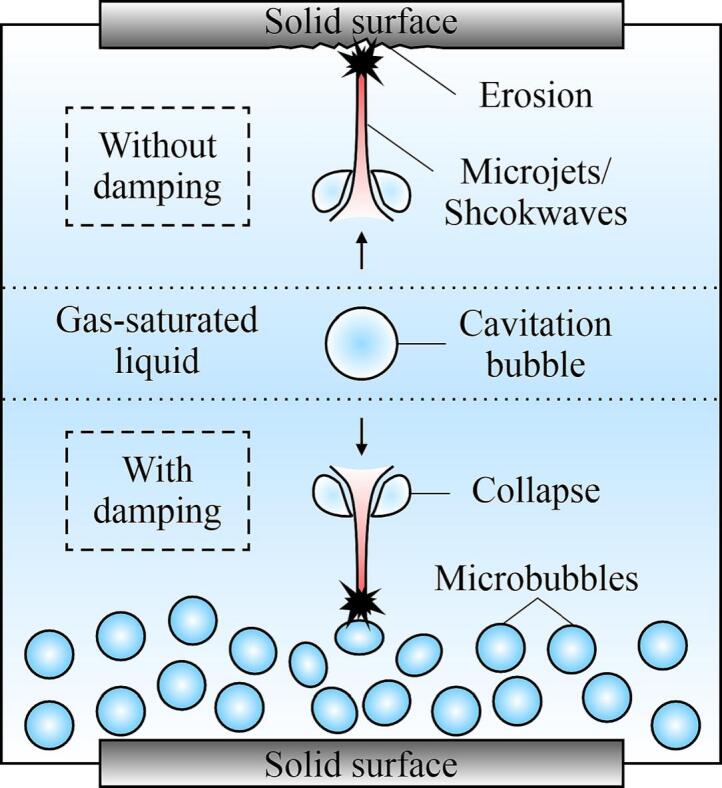
Schematic of mechanisms without (upper part) and with (lower part) microbubbles damping the impact of microjets or shockwaves.

Fig. 10 shows the damping effect of the MBs near solid surfaces. It was observed that CBs would collapse asymmetrically near solid surfaces [63]. Microjets and shockwaves can form because of the implosion of CBs and can impact solid surfaces. In the absence of MBs, a direct impact occurs between the CBs and surfaces, which intensifies CE. In contrast, if MBs exist near a solid surface, the microjets and shockwaves towards the solid surface will first impact the MBs, which are then compressed and absorb the impact energy, thereby reducing the impact intensity on solid surfaces.

When not compressed, MBs were assumed to be spherical with an initial diameter of *d*_0_. MBs are compressed after they absorb the impact energy and convert it into potential energy. The energy dissipation of the microjets and shockwaves is equal to the potential energy change calculated from the change in bubble size. Regardless of whether the MB breaks down, the critical diameter of the MB that cannot be further compressed is denoted as *d*_c_. The compression process is assumed to be adiabatic, and thus, *pV ^γ^* is constant, where *p*, *V* and *γ* represent the pressure inside the MB, volume of the MB, and adiabatic index of the gas (*γ* = 1.4 for air), respectively. Therefore, the work done to compress a single MB radially (from initial volume *V*_0_ to critical volume *V*_c_) in an adiabatic manner is given by

(13) $$\Delta W=-\int_{V_{0}}^{V_{\text{c}}}p\text{d}V=-\int_{V_{0}}^{V_{\text{c}}}\frac{p_{0}V_{0}^{\gamma }}{V^{\gamma }}\text{d}V=\frac{\pi p_{0}d_{0}^{3}}{6\left(\gamma -1\right)}\left[{\left(\frac{d_{0}}{d_{\text{c}}}\right)}^{3\left(\gamma -1\right)}-1\right],$$

where *p*_0_ represent the interior pressure of the MB before compression.

Assuming that all the decreased dissolved air could form MBs, whose number and total volume are denoted as *N*_b_ and *V*_b_, respectively, the maximum work required to compress all these MBs, which represents their potential to alleviate erosion, is given by

(14) $$W_{\max}=\Sigma \Delta W\approx N_{\text{b}}\Delta W=\frac{V_{\text{b}}p_{0}}{\gamma -1}\left[{\left(\frac{d_{0}}{d_{\text{c}}}\right)}^{3\left(\gamma -1\right)}-1\right].$$

It is evident that *W*_max_ depends on *V*_b_ rather than the volume of individual bubbles. *V*_b_ can be approximated using the change in dissolved air content, i.e. *V*_b_ ≈ *δφ*_DO_, where *δ* is a parameter calculated as (liquid volume × (saturated DA/saturated DO) × change rate of DA) / MB density. The liquid volume is ∼ 0.8 L. Due to the saturated N_2_ and O_2_ in water at 25 °C equal to 15 and 8 mg·L^–1^, respectively, *φ*_DA_ ≈ 23*φ*_DO_/9. The MB density is taken as the air density, i.e., 1.2 g·L^–1^. The change rate of DO was ∼10 % (as detailed in Section 2.2). Therefore, *δ* ≈ 1.9 × 10^–4^ L^2^·mg^−1^.

To calculate *W*_max_, we assume that the MB is compressed to half its initial diameter, i.e., *d*_0_/*d*_c_ = 2. According to the Young–Laplace equation, *p*_0_ = *p*_l_ + 4*σ*/*d*_0_ ≈ 0.4 kPa, in which *p*_l_ represents the exterior pressure of the MB (equal to the liquid pressure near the specimen surface) and *σ =* 0.072 J·m^−2^ represents the surface tension coefficient of water at 25 °C. Thus, when *α* > 1,

(15) $$W_{\max}\left(\text{in J}\right)\approx 3.3\times 10^{-4}\alpha .$$

For instance, in Case 10 (*φ*_DO_ = 18.30 mg·L^–1^) with *α ≈* 220 %, *W*_max_ ≈ 726 μJ. The kinetic energy of the microjet was reported to be ∼10 μJ [64], which can be sufficiently absorbed by the MBs formed from supersaturated DA. However, it is important to note that not all MBs can effectively damp the impact of microjets or shockwaves, so that CE still occurred. Furthermore, this model will be improved after specific research on *N*_b_ and *V*_b_. It follows logically that an increase in *α* during sonication will lead to a denser aggregation of MBs near the specimen surface, resulting in increased *W*_max_ and augmented dissipation of impact energy. After the impact is damped by these MBs, the force exerted on the specimen surface will be mitigated, thereby alleviating the CE.

Energy dissipation caused by MBs is related to air entrainment against CE in hydraulic engineering, with numerous studies confirming that small quantities of entrained air bubbles can significantly alleviate or even eliminate CE [20], [21], [22], [23], despite the increasing nuclei. However, the MBs formed in our study were fewer than those entrained in high-speed flows. Therefore, only a slight CE alleviation, rather than a complete elimination, was observed in the supersaturated solutions (Fig. 4b), even with *α* exceeding 2 (*φ*_DO_ = 18.30 mg·L^–1^). Moreover, the alleviation differed from the inhibiting effect of MBs on sonochemistry caused by ultrasound attenuation [48], as erosion primarily results from the impact of shockwaves and microjets near the specimen surfaces, rather than from all cavitation bubbles distributed throughout the liquids. This MB-induced alleviation was also different from that caused by the few nuclei or the gaseous cavitation mentioned above, as evidenced by the scarcity of MBs in undersaturated solutions and the eroded surfaces (Fig. 6c–f). This suggests varying dominant regimes based on *φ*_DA_, even with similar mass loss.

### 3.5 Regimes determining CE affected by DA

**Fig. 11:**
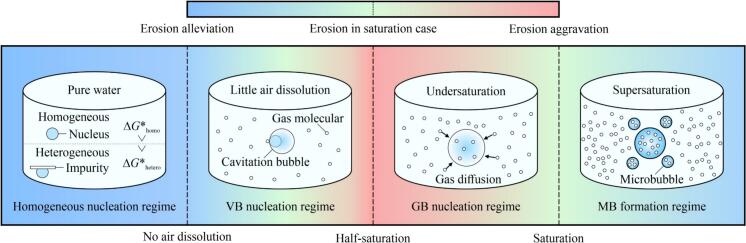
Conceptual classification of regimes influencing ultrasonic cavitation erosion in aqueous solutions containing dissolved air. Darker red and blue shades represent increasing aggravation and alleviation of cavitation erosion, respectively. Green shade represents minimal change in mass loss compared to the saturation condition. ΔG*_homo_ and ΔG*_hetero_ represent the free energy barriers for homogeneous and heterogeneous nucleation, respectively. (For interpretation of the references to colour in this figure legend, the reader is referred to the web version of this article.)

Based on the foregoing discussion, we proposed a classification of the regimes that affect the ultrasonic CE in aqueous solutions containing DA (Fig. 11). This classification identifies four regimes: VB nucleation, GB nucleation, MB formation, and a hypothesized homogeneous nucleation regime with no DA.

The CE under saturation conditions was set as the benchmark. In the MB formation regime in supersaturation, increasing *φ*_DA_ alleviates CE caused by MBs formed because of ultrasonic degasification. Under undersaturation conditions, the CE is influenced by one of the three other regimes. In the range above half-saturation but below saturation, GBs formed because of gas diffusion will not condense and collapse, while the increasing amount of non-condensable gas in CBs decreases the rate of CB collapse. Therefore, CE will be aggravated as *φ*_DA_ decreases between half-saturation and saturation, which is defined as the GB nucleation regime. Our findings suggested that the most severe CE occurred at half-saturation. Further degassing can reduce GB formation and gas molecule diffusion into CBs. Further, minimal amounts of DA can act as nuclei, contributing to the formation of VBs that can generate erosive microjets and shockwaves. This characterizes the VB nucleation regime. Our previous research on the sonochemical effects and CE in solid particle suspensions and aqueous solutions with moderate MB addition, supported the VB nucleation regime [5], [8], [48]. In addition, we propose a homogeneous nucleation regime in which CE is negligible because of the ultrahigh cavitation thresholds. Although not directly observed in our experiments in view of the lack of completely pure water, this regime also highlights the effect of DA on cavitation thresholds and nucleation.

These four regimes clarify CE in an aqueous solution containing DA, with one predominating at a specific *φ*_DA_. Changes in *φ*_DA_ will gradually shift the dominant regime. For example, under supersaturation conditions, both GB nucleation and MB formation may coexist, with the latter dominating because of greater alleviation of CE. Further, the different types of dissolved gases exhibited distinct regimes. Some research found that the effect of dissolved CO_2_ (more soluble than air in water) on CE exhibited a different pattern: increasing CO_2_ initially decreased CE, then increased it, followed by another decrease [65]. This highlights the necessity for further clarification of the underlying regimes of CE in solutions containing different gases and liquids. Such clarification can facilitate the prediction of cavitation intensity and CE in dissolved gas–liquid two-phase flows and the application of ultrasonic wastewater treatment by reducing the horn-tip CE while simultaneously increasing sonochemical efficiency.

## 4 Conclusions

Based on ultrasonic CE experiments in aqueous solutions, the effect of *φ*_DA_ (*φ*_DO_ = 1.18–18.30 mg·L^–1^) on CE was analyzed, and four regimes were proposed to determine whether CE would be aggravated or alleviated.

The main findings are summarized as follows:

a) When *φ*_DO_ increased from 1.18 to 18.30 mg·L^–1^, the mass loss of specimens after 300-min exposure initially increased and then decreased. Compared with the saturation case, supersaturation alleviated CE, while degassing initially aggravated CE, peaking at *φ*_DO_ = 4.00 mg·L^–1^ (the half-saturation case), after which further degassing alleviated CE.
b) In supersaturated solutions, MB formation induced by excess DA during ultrasonication contributed to CE alleviation. When impacted by microjets or shockwaves, these MBs cover the specimen surface and dissipate the impact energy. An increase in supersaturation leads to larger MBs, resulting in greater energy dissipation. Therefore, weaker microjets and shockwaves can impact the specimen surface, alleviating CE.
c) In undersaturated solutions, varying the nucleation rates of the VBs and GBs affected CE. Adding DA to the solutions provides nucleation sites, lowers the energy barrier, and accelerates VB nucleation, thereby aggravating CE when DA is below half-saturation. Further increase in *φ*_DA_ increases GB nucleation rate and non-condensable gas in CBs, which can decrease the collapse rate of CBs and the energy of microjets or shockwaves. The increasing proportion of gaseous cavitation will alleviate CE.

This study highlights the significant role of DA in CE and offers a conceptual classification of regimes to predict CE in dissolved gas–liquid two-phase flow systems, including CE of horn tips in ultrasonic wastewater treatment and damage to hydraulic components. Future research should validate these regimes using more detailed theoretical models and develop multi-phase flow numerical models incorporating non-equilibrium air dissolution effects for more direct CE predictions.

Additionally, this study focuses on the effect of dissolved air under low-frequency ultrasound, which is typically used for sonochemistry. Further investigating the effects of various dissolved gases (e.g., noble gases and highly soluble gases), along with different ultrasonic frequencies (e.g., low: 20–100 kHz, intermediate: 100 kHz–1 MHz, and high: 1–10 MHz) and powers, will enhance the understanding of CE in these systems and improve the application of dissolved gas in wastewater treatment.

## CRediT authorship contribution statement

**Dingkang Xia:** Writing – original draft, Visualization, Methodology, Investigation, Formal analysis, Data curation, Conceptualization. **Jianhua Wu:** Supervision. **Kunpeng Su:** Writing – review & editing, Funding acquisition.

## Declaration of competing interest

The authors declare that they have no known competing financial interests or personal relationships that could have appeared to influence the work reported in this paper.
